# Robust and Multi-Functional Electrically Responsive Gold/Polydopamine-Coated Liquid Crystalline Elastomer Artificial Muscles

**DOI:** 10.3390/nano15211658

**Published:** 2025-10-31

**Authors:** Joshua C. Ince, Setareh Elyasi, Alan R. Duffy, Nisa V. Salim

**Affiliations:** 1 School of Engineering, Swinburne University of Technology, Hawthorn, Melbourne, VIC 3122, Australia; 2 Centre for Astronomy and Supercomputing, Swinburne University of Technology, Hawthorn, Melbourne, VIC 3122, Australia

**Keywords:** LCE, multi-functional, strain sensing, joule heating, artificial muscle

## Abstract

Applying thin electrically conductive coatings to Liquid Crystalline Elastomers (LCEs) is an effective way of functionalizing two-way shape memory polymers with the ability to respond to electrical currents. However, achieving robust adhesion between a given electrically conductive coating and the surface of LCEs can be challenging. This can limit the functionality, performance, and potential applications of these materials. This work describes a facile method to develop electrically responsive Liquid Crystalline Elastomer polymeric artificial muscles with strain-sensing, self-actuation-sensing, and joule-heating features. In this work, the effect of treating LCEs with polydopamine (PDA) prior to functionalizing the LCE with an electrically conductive gold-sputtered coating was explored. The findings confirmed that the PDA treatment considerably improved the adhesion of the gold sputter coating to the LCEs, thereby leading to the fabrication of multi-functional strain-sensing, electrically conductive, and electro-responsive LCEs.

## 1. Introduction

Natural muscles showcase impressive abilities to produce mechanical work, enabling movements like running, climbing, swimming, and flying. There are several candidate technologies for producing artificial muscles, and while these can deform, converting certain stimuli into displacement and mechanical work, they have yet to fully replicate the comprehensive capabilities of biological muscles. Material actuators are one approach to realizing artificial muscles. Material actuators are materials that exhibit a change in shape in response to a given stimulus—they have recently attracted widespread scientific interest. A diverse range of materials and design strategies have been explored to produce material actuators with various actuation types, i.e., bending, curling, rolling, and jumping [1]. In soft robotics, an ideal artificial muscle would mimic the functionalities and performance of natural muscles, i.e., operate with a certain level of freedom, agility, and flexibility, so that heavy and bulky classical mechanical actuators employed in classical robotics can be replaced with lightweight and low-profile flexible material actuators [2]. While material actuators can be metal- or ceramic-based, perhaps the most explored class that closely matches the functionalities and properties of natural muscles is polymer actuators. In contrast to alternative material actuators, polymer actuators are easily prepared, affordable, and elastic and in some cases can self-heal [3]. They also offer intrinsic low masses and high actuation stresses [4].

Polymer actuators are formally known as shape-changing polymers (SCPs); they are a class of polymers that change shape when appropriately stimulated [5,6,7,8,9]. Liquid Crystalline Elastomers (LCEs) are an elastomeric subclass of SCPs that stand out due to their potential to serve as artificial muscles, which arises from their distinctive mechanical properties, unique polymeric structure, and thermal phase transitions [10,11,12,13,14,15]. LCEs may have applications in several areas, including energy dissipation [16,17], bio-scaffolds [18], and switchable reflectivity [19,20,21,22]. However, they are most researched for their impressive shape-changing properties. Broadly speaking, elastomers that do not exhibit shape-changing properties are still intriguing materials to equip with additional functionalities. Their molecular structures and arising mechanical properties allow for these materials to be stretched and strained without permanently deforming their shapes. Typically, elastomers are insulative, limiting their sensing capabilities. However, if they can be rendered electrically conductive, they can find applications in areas such as strain sensing or flexible heaters in a rather straightforward manner. Their soft and deformable nature, coupled with the aforementioned endowed electrical conductivity, results in a material that can either exhibit a measurable change in electrical resistance when strained or a material that can be strained and flexed while still converting electrical energy into thermal energy.

While numerous studies have emphasized the importance of LCEs as artificial muscle candidates, there are several functionalities that natural muscles harbor that LCEs typically do not. One such crucial functionality is strain sensing. The ability to sense strain in natural skeletal muscles aids in preventing muscular overexertion and enables a feedback loop critical for smooth and dynamic movement [23,24]. The integration of functionalities like strain sensing into LCEs represents a significant advancement in artificial muscle research. Several research groups have explored the concept of strain-sensing LCEs. Devesh and colleagues [25] investigated the optical correlation properties of isotropic LCEs in response to induced strain. However, isotropic LCEs do not actuate. Furthermore, LCEs with optical strain-sensing properties are not ideal for artificial muscles since a system of measuring the change in optical properties would be required to measure the strain. A simpler approach is to render the LCE conductive and to sense the strain piezo-resistively.

Ford et al. [26] introduced a notable piezo-resistive strain-sensing LCE by incorporating gallium microspheres into LCEs, achieving multi-functional strain-sensing gallium/LCE composite materials that demonstrated piezo-resistance and conductivity, marking a significant breakthrough in flexible actuation research. At the time, this innovation showcased one of the most multi-functional LCEs reported, but certain limitations arose, such as a degradation in the mechanical properties and the need to maintain a temperature above 30 °C for the material to remain elastic.

Recently, a concept has emerged in the literature to combat these constraints. The approach is to coat only the surface of LCEs to be electrically conductive, rather than functionalizing the entire matrix [27,28]. This way, the mechanical properties of the LCE are minimally impacted. Typically, these methods explore electrically conductive LCE coatings as ways of triggering the actuation of the LCE via the phenomenon of joule heating. Sun et al. [27] reported a method for coating LCE fibers with conductive liquid metal. When an electrical current was applied to the fibers, the fibers were able to actuate in sub-second time frames. Wu et al. [28] also reported a method for producing conductive and photothermal PDA/MXene-coated LCE fibers that could also actuate in >1 s. However, few works explore the strain-sensing and multi-functionality components of electrically conductive LCEs.

In a previous study, our group reported a solution-based method for coating LCEs with silver nanoparticles (Ag-NPs) to yield multi-functional strain-sensing and electro-responsive LCEs [29]. However, this method was time-consuming, and we found evidence to suggest that the method damaged the mechanical integrity of the LCE. Fortunately, there is a simple and fast method of applying thin conductive coatings to materials that is still relatively unexplored in the field of LCE research: sputter coating.

Sputter coating is an accessible and efficient means of depositing nanometer-thick metal layers onto a substrate [30,31,32,33]. It has even been directly employed to coat elastic polymers with thin conductive coatings to yield effective strain sensors [34,35]. Sputter coating LCEs has even been recently explored by Wang et al. [36] as a means of rendering the LCE joule-heating/electro-responsive. The resultant samples were capable of rapidly actuating in response to electrical currents. However, to prevent the Au layer from cracking and delaminating during the actuation process, the authors had to first stretch the LCE and hold it in its strained state while it was sputter-coated. On the one hand, this prevented the Au layer from cracking and delaminating during the actuation process; however, on the other hand, this approach prevented the samples from being able to piezo-resistively sense strain.

For surface-functionalized piezo-resistive elastomers to sense strain, they are often first crack-trained [37,38]. Crack training involves pre-straining and un-straining the elastomer so that micro-scale cracks are formed in the sputter-coated layer. This way, when the elastomer is strained, micro-cracks are consistently separated, resulting in a consistent increase in resistance that can be measured. However, because LCEs have low surface energies, it can be challenging to achieve a sputter-coated LCE that can be crack-trained without the sputter-coated layer delaminating and flaking off.

PDA is a well-researched biopolymer that is in part known for its universally adhesive properties. PDA coatings have been successfully applied to many low-surface-energy materials, such as fluoropolymers and silicone-based polymers. However, in addition, PDA is also highly explored for its ability to bind nanomaterials of all sorts to various substrates, rendering it a uniquely useful material when it comes to improving the adhesion between two materials with low adherence to one another. In our work, where we coated LCEs in Ag-NPs, we even employed PDA to anchor the particles to the LCE surface. We observed that PDA coating robustly adhered the Ag-NPs to the LCE [29]. Hence, we were interested in exploring whether PDA could improve the adhesion of Au sputter coating to LCEs.

We found that PDA-treating LCEs prior to Au sputter coating greatly improved the adhesion of the gold-sputtered layer to LCEs. Concurrently, the PDA treatment also greatly improved the sensing properties of the Au-coated LCEs. As a result of the PDA treatment, the samples were robust enough to endure cyclical straining and actuating the samples without the gold coating delaminating or the electrical conductivity considerably changing. The Au-PDA-LCE samples were demonstrated to be capable of sensing strain, sensing their own actuation strokes, and being stimulated into actuation with electrical currents via the phenomenon of joule heating. Our findings report a straightforward and economic method of producing robust multi-functional electrically responsive LCE flexible actuators/artificial muscles.

## 2. Materials and Methods

### 2.1. Materials

RM257 was purchased from Suzhou Xiaoli Pharmatech Co., Ltd., Suzhou, China at a purity of 98.9%. 2,2-(ethylenedioxy) diethanethiol (EDDET), pentaerythritol tetrakis(3-mercaptopropionate) (PETMP), dipropyl amine (DPA), (2-hydroxyethoxy)-2-methylpropiophenone (HHMP), and Toluene (TOL) were all purchased from Sigma Aldrich Bayswater, Australia.

### 2.2. Production of LCE Polymer Actuator

Synthesis of linear main-chain liquid crystal polymer was conducted in accordance with the method outlined by Yackaki et al. and Saed et al. [39,40]. In a 30 mL vial, 4 g of RM257 was dissolved in 1.6 g of TOL in an oil bath set to 80 °C. Next, after the solution was cooled to room temperature, 0.217 g of PETMP and 0.9157 g of EDDET were added to the solution and mixed with a vortex mixer. If recrystallization of RM257 occurred, the vial was placed back into the 80 °C oil bath until the monomer redissolved. Then, 0.0257 g of HHMP was added to the solution, followed by 0.568 g of a diluted catalyst solution, DPA (DPA was diluted with TOL at a ratio of 1:50). To remove air bubbles, the monomer was placed in the vacuum chamber at 508 mmHg for 1 min before being immediately injected between two 150 × 120 mm glass plates that had been sprayed with a commercially available superhydrophobic coating. Glass spacers, 1 mm thick, were used to control the thickness of the films prior to alignment. After being left to polymerize for 24 h, the samples were placed in a drying oven set to 80 °C for 24 h. The isotropic LCEs were removed from between the two plates, cut into 15 × 30 mm strips, stretched to 100% strain, and cured for 15 min with a 230 V/0.17 A ENF-240C/FE Spectroline UV lamp (New York, NY, USA) set to 365 nm.

### 2.3. Polydopamine Coated LCEs

LCE PDA coating was completed in accordance with the method outlined in our previous work [29]. A 0.1 M 70:30 *v*/*v*% EtOH:H_2_O tris-HCl buffer solution was prepared before adjusting the pH to 8.5 via dropwise addition of a 10 wv% HCL solution. While variables such as dopamine concentration and pH were not optimized to control the deposition of PDA onto the LCEs, it is important to note that in our prior work, we already developed a modified PDA deposition method for achieving a uniform and robustly adhered coating of PDA to the LCEs via the addition of 30 wv% ethanol to the dopamine coating solution. Next, the cut LCE films were submerged in the solution before 2 mg/mL of dopamine–HCl was added to the solution. The samples were gently mixed on a magnetic stirrer hotplate at room temperature for 12 h. After 12 h, the samples were then removed from the PDA coating solution and washed with DI and then ethanol before being left to dry overnight at room temperature.

### 2.4. Production of Au-Sputter-Coated LCEs and Crack Training

Both the control and PDA-coated LCEs were sputter-coated with gold using a Q150R ES Plus gold sputter-coating unit (Sydney, Australia) for 60 s on each side of the samples (equivalent to a 40 nm coating on each side of the samples based on the manufacturer’s specifications). Argon was used as an inert process gas. The sputtering current was 40 mA. Finally, once coated in gold, the samples were crack-trained. This was completed by loading the samples into a TA Instruments DHR-1 Dynamic Mechanical Analyzer, Rydalmere, Australia. The samples were cyclically strained and relaxed 100 times with an applied strain of 30% and a strain rate of 1%s^−1^.

### 2.5. Characterization

Scanning electron microscopy (SEM) images were captured on a Zeiss Gemini SUPRA-40 Scanning Electron Microscope, Sydney, Australia. DMA was completed with the same TA Instruments DHR-1 DMA used to crack-train the samples. Strain-sensing and self-actuation-sensing characterizations were completed by coupling a Keysight Digital Acquisition device (DAQ) (Mulgrave, Australia) in 2-point Ohmic resistivity mode with the DMA. Figure S1 details the employed setup. For the strain-sensing characterizations, samples were cyclically strained and relaxed at 25 °C with a constant rate of 1%/s. To obtain self-actuation sensing data, an iso-strain program was employed on the DMA. Using an in-house furnace/cooler system, the temperature was cyclically heated to 90 °C and cooled to 25 °C to induce the actuation/relaxation of the samples while the DMA measured the actuation stress and the DAQ logged the sample resistance.

### 2.6. Adhesion Testing

Four different approaches were used to assess the adhesion of the gold layer to the samples. The first approach was to assess whether the gold layer remained adhered to the samples after the samples were cyclically strained and actuated. The samples were strained (30%) 100 times and actuated 100 times using a DMA before being assessed via SEM. The second approach (the tape-pull test) involved applying adhesive tape to the sample’s surfaces. The tape was subsequently pulled off, and the adhesion of the gold-sputter-coated layer on the samples was optically assessed. The electrical resistance was also measured before and after conducting the tape-pull test to assess the effect of the test on the samples’ conductivity. The third approach was to conduct heat-quench testing. This involved cutting the samples into 20 × 10 mm rectangles and placing them into an oven set to 200 °C for 1 h. Immediately after, the samples were plunged into an ice bath to rapidly heat-quench them. After drying, the surfaces of the samples were observed via SEM. The final approach was to conduct pull-off testing. To do so, samples were cut into 10 × 10 mm squares. Next, a series of ABS dollies was 3D-printed. Then, using epoxy glue, the dollies were adhered to each side of the sample. The dollies were then loaded into the DMA. In tensile mode, the dollies were then pulled apart, and the force required to delaminate the gold-sputter-coated layer from the sample was measured.

## 3. Results and Discussion

**Figure 1:**
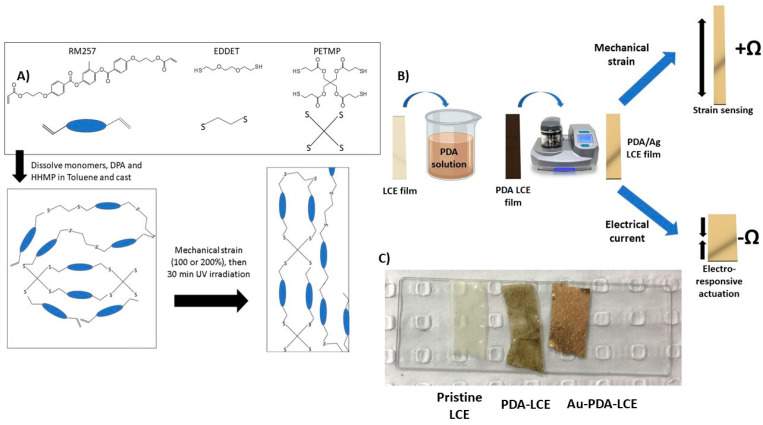
Schematic synthesis and fabrication of Au-PDA-LCEs. (**A**) Chemical synthesis and alignment of LCE; (**B**) schematic PDA and Au sputter coating of LCEs; (**C**) from left to right: pristine LCE, PDA-LCE, and Au-PDA-LCE.

Figure 1 depicts the schematic synthesis and fabrication of the Au-PDA-LCEs explored in this study. After successfully being coated in PDA, the LCEs changed from being a pale transparent yellow to a dark brown. Finally, after sputter coating the samples, the samples were visibly rendered gold in appearance (see Figure 1C) and were validated as being conductive using a multimeter.

**Figure 2:**
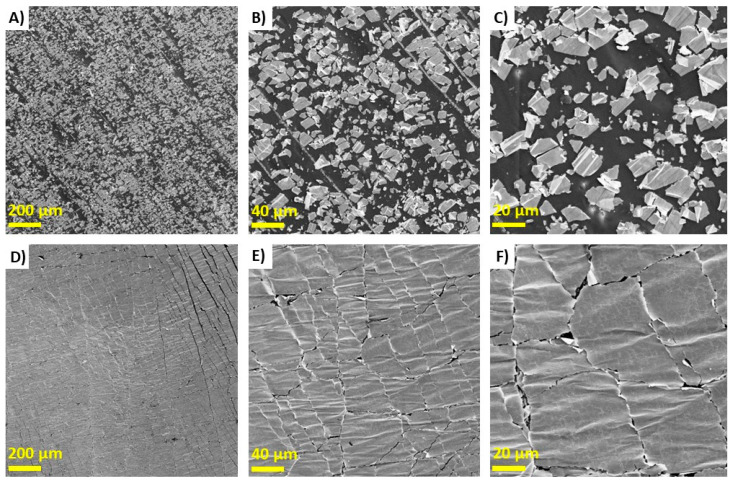
SEM images of control Au-LCE at ×50, ×100, and ×1000 magnification (**A**–**C**) vs. Au-PDA-LCE at ×50, ×100, and ×1000 magnification (**D**–**F**) after being crack trained.

After establishing that we had successfully coated the surface of both the control Au-LCE and the Au-PDA-LCE with gold, we assessed the adhesion of the gold layers to the samples. All of the adhesion tests demonstrated that the PDA treatment considerably improved the adhesion of the Au layer to the LCE. As seen in Figure 2, after the control and PDA-coated LCEs were cyclically strained and actuated 100 times, SEM analysis revealed that the Au-sputter-coated layer was still well adhered to the surface of the PDA-coated LCE. Conversely, for the control sample, the gold layer was considerably delaminated. For the PDA-coated sample, despite the cyclical straining and actuation of the sample, the surface of the LCE was completely covered in gold micro-flakes ranging from 20 to 150 µm, while for the control sample, the pristine LCE surface was exposed, and the micro-flakes were much smaller (>30 µm).

**Figure 3:**
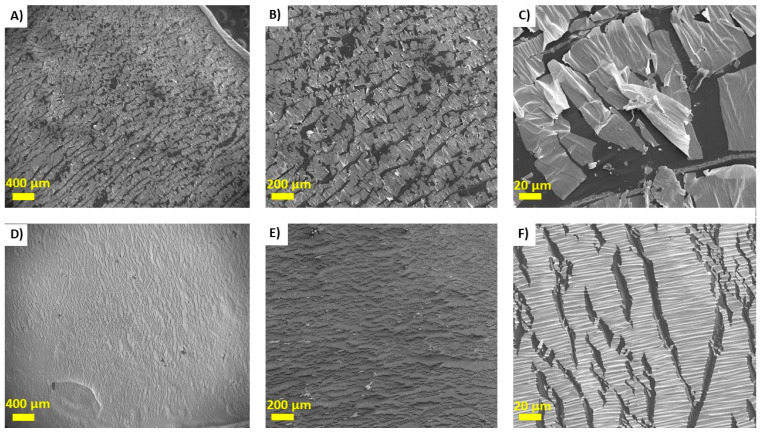
SEM images for control Au-LCEs at ×50, ×100, and ×1000 magnification (**A**–**C**) and Au-PDA-LCEs at ×50, ×100, and ×1000 magnification (**D**–**F**) after heat-quenching.

The heat-quench testing also showed similar results. As seen in Figure 3, after heat-quenching, the gold layer had considerably cracked and delaminated from the control LCE surface. However, for the PDA-coated LCE, the gold layer was observably cracked, but no signs of delamination were observed.

**Figure 4:**
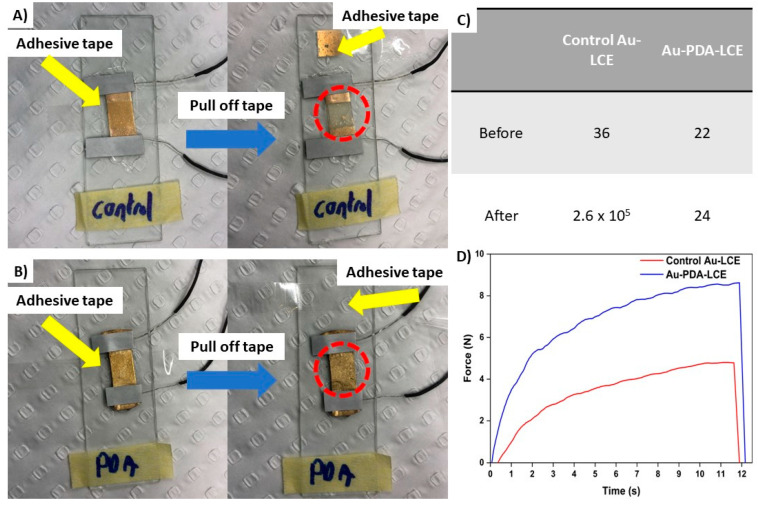
Depicting results of tape-pull testing. (**A**) Delamination of the Au-layer from the control LCE due to tape-pull testing, (**B**) adhesion of the Au-layer to the PDA-coated LCE despite tape-pull testing, (**C**) improved adhesion of the Au layer to the PDA-coated LCE compared to the control LCE, (**D**) electrical resistance of samples after tape-pull testing.

The tape-pull test results also confirmed that the PDA treatment improved the adhesion of the Au-layer. As seen in Figure 4A,B, when the tape was pulled off the control Au-LCE, the Au layer almost completely delaminated. Only a trace amount of gold remained on the surface. Conversely, for the PDA-coated LCE, the Au layer remained completely adhered to the PDA-LCE. The electrical resistance before and after the tape-pull test can also be seen in Figure 4C. For the control Au-LCE, the resistance increased from 36 to 2.6 × 10^5^ Ω^−1^. For the Au-PDA-LCE sample, the resistance marginally increased from 21 to 24 Ω cm^−1^.

Finally, the results of the pull-off test quantitatively demonstrate the improved adhesion of the Au layer to the PDA-coated LCE. As seen in Figure 4D, the force required to pull the Au layer from the PDA-coated LCE was over double that of what was required for the control Au-LCE. Both samples demonstrated adhesive fractures, with the Au layers being pulled off the LCEs.

The improved adhesion of the gold layer sputtered on the PDA-coated LCEs is likely due to the formation of intermetallic and complex bonds between the catecholamine functional groups present in the PDA structure. As discussed in Lee et al. [41], dopamine, being a catecholamine, can bond to various substrates, including noble metals, via the formation of both intermetallic bonds and complex bonds. Pierpoint et al. [42] discussed how Au atoms bond to catechol via weak intermetallic bonds, while Dalsin et al. also conducted XPS analysis on catechol-coated gold substrates and found evidence of AuOC^+^, AuO_2_C_2_^+^, and AuO_2_C_6_H_5_^+^ fragments, definitively concluding that catechol can form complex bonds with solid gold. Hence, while we cannot say which of the two mechanisms resulted in the improved adhesion, or if the improved adhesion was a result of a combination of the two, which was the dominant mechanism, we can assert that the mechanism behind the improved adhesion of gold to PDA is due to the formation of weak intermetallic bonds and complex bonds between the PDA and the sputter Au coating.

**Figure 5:**
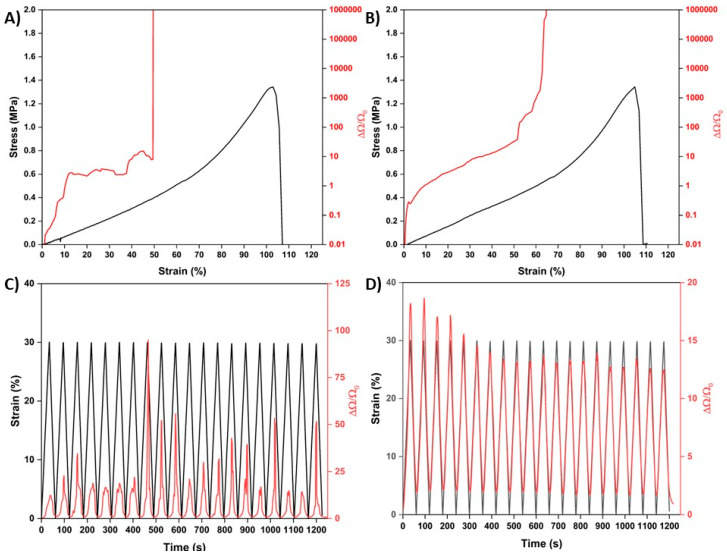
(**A**,**B**) Stress–strain curves and resultant gain factor signals (ΔΩ/Ω_0_) of the control Au-LCE sample and the Au-PDA-LCE samples, respectively. (**C**,**D**) Cyclical straining and resultant gain factor signals (ΔΩ/Ω_0_) of the control Au-LCE sample and the Au-PDA-LCE samples, respectively.

After establishing that the PDA treatment improved the adhesion of the Au layer to the LCE, the sensing capabilities of the samples were assessed. Figure 5A,B show how the gain factor (ΔΩ/Ω_0_) changed as the samples were mechanically strained. The PDA treatment notably decreased the noise in the ΔΩ/Ω_0_ signal. As seen in Figure 5A, for the control Au-LCE sample, the ΔΩ/Ω_0_ increased proportionally with strain; however, the signal was noisy and unstable. For the Au-PDA-LCE sample (Figure 5B), the ΔΩ/Ω_0_ signal had less noise, was more stable, and was smoother. The PDA treatment also appeared to improve the sensing range. For the control sample, at 50% strain, the ΔΩ/Ω_0_ increased dramatically beyond the sensing capability of the DAQ. However, for the PDA-treated sample, this did not occur until 60%.

The samples’ ability to cyclically sense strain was also assessed. As seen in Figure 5C,D, the control sample was able to track whether it was being strained or not; each peak of the ΔΩ/Ω_0_ plot matched the peaks for the cyclically applied strain plot. However, the ΔΩ/Ω_0_ signal was inconsistent and noisy. In contrast, for the PDA-treated sample (Figure 4B,D), the ΔΩ/Ω_0_ signal was markedly less noisy and tracked the cyclically applied mechanical strain closely. Despite the samples being crack-trained, it still took five strain cycles for the ΔΩ/Ω_0_ signal sample Au-PDA-LCE to stabilize. After passing five cycles, the average of the ΔΩ/Ω_0_ peak heights for the control Au-LCE was 35.31 ± 21.93. For the Au-PDA-LCE sample, the average was 13.22 ± 0.57, which is a considerable improvement. We also tested the long-term cyclical strain-sensing capabilities of the Au-PDA-LCEs by cyclically straining the sample 100 times. The results and discussion can be seen in the supplementary information document and Figure S2.

It is important to emphasize that the strain-sensing testing completed in this work employed two-point probe testing. This limited the strength of the obtained results, as we could not measure the sheet resistance of the samples during straining, which is an important parameter to measure when formally assessing strain sensors. However, it should also be emphasized that the purpose of this study was not to develop a high-performance strain sensor but rather to explore the effects of treating LCEs with PDA prior to sputtering the LCEs with gold and to assess if this had an effect on the various functions that we hypothesized the samples would harbor.

Moreover, two-point probe testing was employed during the strain sensing; it is difficult to quantitatively compare the results of the Au-PDA-LCEs as strain sensors to the performance of other strain sensors reported in the literature. However, we can assert that the strain-sensing functionality of the Au-PDA-LCEs is sub-optimal when compared to effective strain sensors recently reported in the literature. Namely, the gain factor is rather small compared to other notable high-performance soft strain sensors. Additionally, while the PDA treatment improved the reliability of the strain-sensing functionality, the samples still did not exhibit accurate or precise strain sensing. This is apparent when assessing the results of the cyclical strain sensing tests. For each strain cycle, which was precisely and consistently applied, there was a reasonably large difference in the gain factor peak heights and an apparent shift in the peak heights as the cyclical testing went on. It should also be noted that this was after the samples had already been crack-trained, indicating that this is not an initial effect that would plateau. This is evidence that while the samples are impressive multi-functional materials (i.e., it is atypical for a single material to exhibit strain-sensing, joule-heating, shape-changing, and self-actuation sensing functionalities), the samples were not ideal strain sensors.

**Figure 6:**
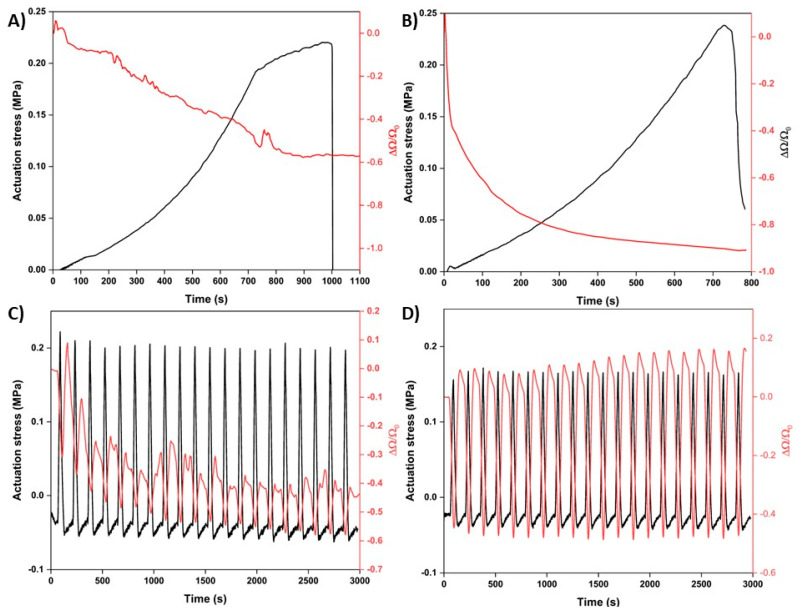
(**A**,**B**) Maximum actuation stress testing and resultant gain factor signals (ΔΩ/Ω_0_) of the control Au-LCE sample and the Au-PDA-LCE samples, respectively. (**C**,**D**) Cyclical actuation testing and resultant gain factor signals (ΔΩ/Ω_0_) of the control Au-LCE sample and the Au-PDA-LCE samples, respectively.

In addition to improving the adhesion and strain-sensing functionality, the PDA treatment also improved the ability of the Au-PDA-LCE sample to sense its own actuation strokes. As seen in Figure 6A,B, when the samples were stimulated to actuate, the ΔΩ/Ω_0_ decreased for both samples. However, for the control sample, the ΔΩ/Ω_0_ signal was again noisy and unstable. For the Au-PDA-LCE sample, the ΔΩ/Ω_0_ signal was considerably smoother and less noisy. The ΔΩ/Ω_0_ also decreased by ~1.5 times more than the control sample. The improvement in the self-actuation sensing due to the PDA treatment was also observable when we cyclically tested this functionality. As seen in Figure 6C,D, compared to the control sample, the ΔΩ/Ω_0_ signal for the PDA-treated sample was smoother, more consistent, and less noisy. The average ΔΩ/Ω_0_ trough value for the control Au-LCE sample was −0.508 ± 0.072, while for the Au-PDA-LCE sample, the value was −0.476 ± 0.009. It is important to note and draw attention to the fact that we did not conduct long-term cyclical actuation testing. Such testing is critical to understanding and assessing the long-term stability of the produced Au-PDA-LCEs during repeated actuation. Further investigations are warranted to assess the electrical properties of the Au-PDA-LCEs after being cyclically actuated on a long-term scale.

**Figure 7:**
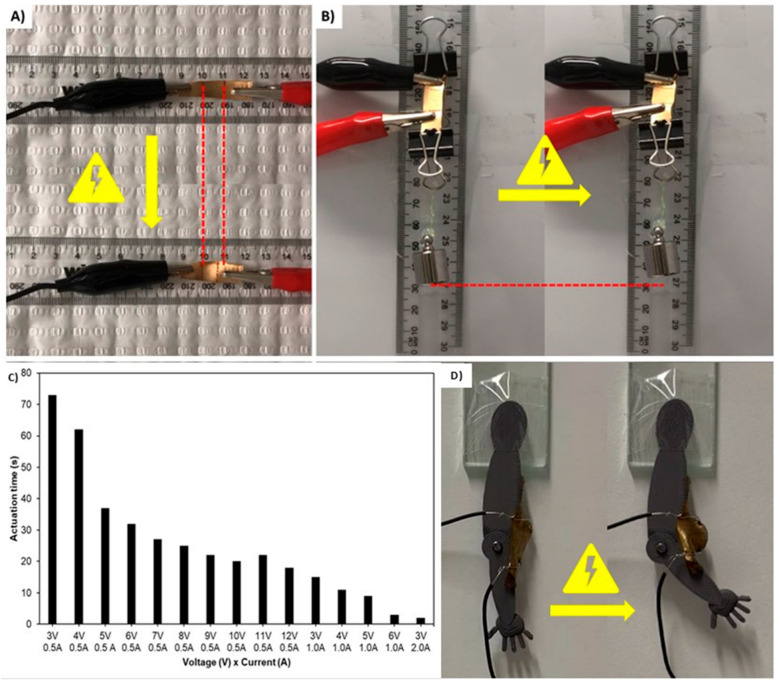
Demonstration of Au-PDA-LCE-45s joule-heating actuation functionality. (**A**) Actuation response of Au-PDA-LCE to an electrical current. (**B**) Au-PDA-LCE demonstrating the ability to perform work by lifting a 10 g weight in response to an electrical current. (**C**) Demonstration of actuation time in response to different current–voltage combinations, (**D**) Au-PDA-LCE driven “arm” moving in response to an electrical current applied to the LCE “muscle”.

In addition to being conductive and strain-sensing, the samples were also capable of electro-responsive actuation via joule heating of the Au-sputtered layer. As depicted in Figure 7, using a DC electrical benchtop power supply set to 2.0 V and 0.5 A, we were able to induce the actuation of the Au-PDA-LCEs. Figure 7A and Video S1 demonstrate the ability of the sample to actuate in response to an electrical current. Furthermore, Figure 7B and Video S2 demonstrate the ability of the sample to produce mechanical work in response to an electrical current by lifting a 10 g weight (~50 times its own mass). As depicted in Figure 7C, a brief experiment was conducted to explore how different DC voltage–current combinations affected the actuation rate. Increasing the power naturally increased the actuation speed. Moreover, the applied current was more correlated with the actuation speed. When a 3 W, 6.0 V, 0.5 A current was applied, the sample took ~32 s to completely actuate. However, when a 3 W, 3 V 1.0 A current was applied, the sample took ~13 s to completely actuate. Moreover, when a 6 W, 6 V 1.0 A current was applied, the sample took ~2 s to completely actuate. This compares with when the power is fixed (6 W), but using 12 V and 0.5 A, the sample took ~16 s to completely actuate. This is consistent with the principle of joule heating, where the power dissipated as heat (P) is equal to the current squared (I^2^), multiplied by the resistance (Ω) (i.e., P = I^2^Ω). Hence, applying equivalent power, but with higher current and lower voltage, leads to more efficient heat generation and, thereby, accelerated heat transfer and a faster actuation rate. While this observation is consistent with the joule heating principle, it is important to specify, as this observation can lead to more effective joule-heating LCE actuators and is not thoroughly discussed in the related literature.

For the final demonstration of the Au-PDA-LCE’s ability to electro-responsively actuate, a piece of Au-PDA-LCE was adhered to a 3D-printed “arm” to serve as an artificial “bicep muscle”. As seen in Figure 7D and in Video S3, when an electrical current was applied to the Au-PDA-LCE, the arm bent due to the actuation of the Au-PDA-LCE bicep. The arm was able to be cyclically actuated via the application of a 3 V, 1.0 A current.

**Figure 8:**
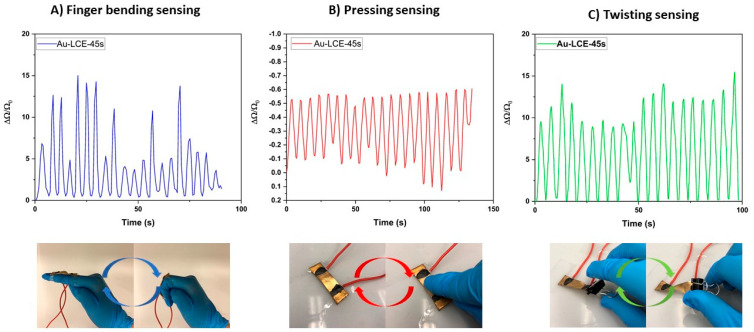
Depicting the ability of sample Au-LCE-45s to sense (**A**) finger bending, (**B**) pressing, and (**C**) twisting.

Finally, to explore the developed sample’s ability to sense movement, three different movement-sensing experiments were conducted. To ensure a good electrical connection between the sample and the DAQ, the electrical wires were adhered to the sample using a conductive adhesive. The wires were then connected to the DAQ, and we commenced the experiments. In one experiment, the sample was mounted onto a gloved human index finger, and then, the finger was cyclically bent and flexed. In the second experiment, the sample was placed on a flat benchtop and repeatedly pressed. Finally, for the third experiment, one end of the sample was mounted on a flat benchtop, while a bull clip was attached to the opposite end of the sample. The sample was then cyclically twisted and untwisted. The results can be seen in Figure 8. For the finger-bending-sensing experiment, the electrical resistance was observed to increase and decrease cyclically in correspondence to the bending and flexing of the finger. This result was unsurprising since this experiment was fundamentally demonstrating the piezo-resistive strain-sensing functionality that had already been validated. However, the sample was also able to sense when it was pressed and twisted. For the pressure-sensing experiment, the electrical resistance decreased when the sample was pressed. This led to a negative ΔΩ/Ω_0_ in response. The sample was also able to successfully sense being twisted. Upon cyclically twisting and untwisting the sample, the electrical resistance increased and decreased in response.

Throughout this series of experiments, a consistent gain factor signal was not obtained when straining, pressing, or twisting the sample. This is likely due to the inconsistent strain/pressure/torsion applied by finger bending, pressing, or twisting, as these stimuli were manually applied. This speculation is supported by the consistency of the gain factor response from the DAQ-coupled DMA strain-sensing characterization.

Our work demonstrates that the PDA treatment of LCEs greatly improved both the adhesion of the gold-sputter-coated layer on the LCE and the piezo-resistive sensing functionality. From our study alone, it is difficult to pinpoint the precise reason for these results because this study was designed to assess the effect of treating LCEs with PDA prior to sputter coating them rather than exploring the mechanism behind the effects. However, several studies have mechanistically explored how PDA can be used to improve the adhesion between polymer–metal interfaces. These findings provide valuable insights into the probable cause of the results obtained in our experiments. Xin et al. [43] conducted molecular dynamics simulations to understand the effect of PDA treatment on the interfacial adhesion between copper and a thermoplastic (polyphenylene sulfide). The authors reported that PDA treatment improved the interfacial energy either due to the formation of hydrogen bonds between the PDA and the copper or due to the improved intermolecular forces arising from the increased surface roughness because of the PDA treatment. Kafkopoulos and others observed similar effects on the adhesion between PDA-treated titanium wires and polymethyl methacrylate-co-methacrylic acid. By conducting pull-out testing, the authors showed that PDA treatment of titanium wires improved the crack propagation force between the titanium wires and polymer interface by a factor of ~6 [44].

**Figure 9:**
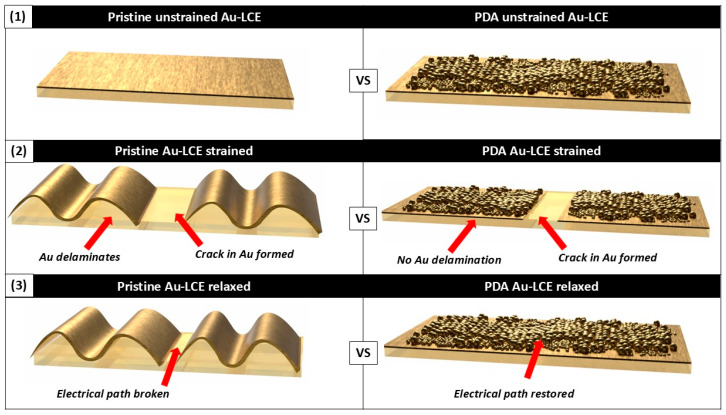
Illustrated proposed mechanism for improved sensing in PDA-treated Au-sputtered LCEs.

Hence, the probable explanation behind the improved adhesion of the Au-sputtered layer and the PDA-coated LCEs is an increase in the interfacial energy and intermolecular forces. As a result of the improved adhesion, when crack-trained, the Au micro-flakes on the PDA-treated LCE did not delaminate and bend (see Figure 9), which was what was observed for the pristine Au-LCE. This resulted in a more precise and stable change in the ΔΩ/Ω_0_ and an overall improvement in the piezo-resistive sensing properties. Regardless of the mechanistic cause, our results clearly demonstrate that adhesion was considerably improved, and, as a result, the sensing performance of the Au-sputter-coated LCEs was improved due to the PDA treatment.

While the outcomes of this work are generally positive (i.e., multi-functional LCEs were produced, the adhesion between the LCE and the Au-sputtered layer greatly improved due to PDA treatment, and the sensing functionalities of the PDA-treated LCEs were significantly improved compared to the control samples), there are still several uncertainties and limitations in this technology. Starting with the uncertainties, the long-term actuation performance of LCEs as polymer actuators or artificial muscles has been studied but not as extensively as it should be if these materials are to find commercial applications as artificial muscles. For instance, there are several works that have demonstrated a negligible loss of actuation performance in LCE actuators even after thousands of actuation cycles. However, operating over the span of a year, it would be reasonable to expect an artificial muscle in a robot to perform tens to even hundreds of thousands of actuation cycles. The reality is that, to date, such long-term testing of LCEs simply has not been performed. While the negligible drop in actuation performance after thousands of actuation cycles is a promising indication that LCEs can maintain long-term actuation performance, without conducting such testing, this will always be uncertain.

Moving on to the limitations of the LCEs reported in this work, the primary limitation is the scalability of the production method. The LCEs in this work are LCE films. To date, there are no known reported methods for scalable mass production of LCE films. Perhaps techniques such as slot-die casting may allow for a roll-to-roll production method, but this is purely speculative at this point. In addition, while our work demonstrates that PDA treatment considerably improves the adhesion and performance of sputtered Au coatings on LCEs, PDA treatment can be difficult to automate and scale because of the reaction dynamics—as dopamine polymerizes into PDA, it is consumed, limiting continuous PDA deposition. Moreover, as PDA particles begin to form, they increase in size and, as a result, have faster sedimentation rates, making it difficult to keep the particulates dispersed as the reaction proceeds and making it difficult to uniformly coat substrates as the reaction proceeds. Again, perhaps techniques such as flow reactors may provide value in achieving continuous PDA deposition, but this is purely speculative. Finally, the technique of sputtering is also a challenging technique to scale and conduct in a continuous manner. There are several commercially available sputtering systems that allow for roll-to-roll magnetron sputtering onto thin films, but these machines do not have high production rates, raising challenges for mass production, which would be necessary for a technology such as LCE-based artificial muscles.

While there are many approaches to rendering LCEs conductive—such as blending conductive additives into LCE matrices—coating the surface of LCEs with conductive materials is an emerging approach that is demonstrably highly effective. As discussed in the Introduction, sputter coating is an attractive method for coating LCEs with thin electrically conductive coatings. However, to the best of our knowledge, no other studies have reported means of improving the adhesion of sputter coatings onto LCEs, which is a critical parameter that should be explored for any technology involving bonding between two different materials.

The principal significance of this work was the improvement in adhesion between the Au-sputtered layer and the LCE owing to the PDA treatment. This is significant, as it validates how PDA’s universal adhesive properties can be harnessed to achieve robust adhesion between materials and LCEs that typically would not bolster good adhesion. There are several materials that are interesting candidates to coat LCEs in, including conductive organic materials and polymers; various metals; and, of course, nanomaterials such as graphene, CNTs, MXenes, and nanoparticles. While the latter candidate materials have recently been somewhat explored, there are few works exploring the formerly mentioned candidate materials. One undisputable reason that few works have explored coating LCEs with materials such as conductive polymers and various metals is that it is challenging to achieve robust adhesion between the materials and LCE surfaces. This work provides a simple and practical method to allow for the exploration of this topic. For example, there are several alternative metals to gold that may be far more interesting candidates for coating LCEs with—silver has a lower thermal conductivity than gold, and it has higher electrical conductivity. This makes silver a superior joule-heating material compared to gold. However, most works focus on electroless deposition of silver onto PDA-coated substrates, including our own previous work: Ince et al. [29]. We predict that this is likely because while many researchers are aware that aqueous silver ions can be complexed and reduced into silver nanoparticles using PDA, few are aware that PDA can adhere directly to solid metals, including sputtered noble metals. Hence, this study provides the framework for warranting investigations into sputtering LCEs with a variety of metals, or possibly even for investigating the effect of coating LCEs with metal alloys to achieve interesting and unique thermal/electrical properties. Furthermore, Table 1 lists and compares several notable LCE works reported in the literature over the past 5 years, including the current presented work.

## 4. Conclusions

This work reported the fabrication of robust strain-sensing, electrically conductive, and electro-responsive LCEs. This was achieved by Au sputter coating PDA-treated LCEs. The PDA treatment was shown to significantly improve both the adhesion of the gold-sputtered layer to the LCE and the strain-sensing functionality of the samples. The Au-PDA-LCE samples were able to accurately piezo-resistively sense applied mechanical strain. They were also able to sense their own actuation strokes and actuate in response to electrical currents via the phenomenon of joule heating. Without PDA treatment, the gold-sputtered layer observably delaminated and flaked off the control Au-LCE samples when it was cyclically actuated and strained. However, for the PDA-treated samples, the Au layer remained adhered to the LCE not only after cyclical straining/actuating but also after a piece of adhesive tape was applied to the sample and pulled off. This work adds to the growing body of literature that is continuously demonstrating the efficacy of LCEs functionalized with conductive coatings to serve as electro-stimulated, multi-functional artificial muscles. Moreover, this work reports critical findings on how the adherence of conductive coatings applied to LCEs can be improved.

## Figures and Tables

**Table 1:** Comparing notable LCE works reported in the literature within the last 5 years, with the work presented in this manuscript (This work **).

Type	Reference	Method Simplicity	Additional Comments	Additional Functionalities	Benefits	Constraints
Joule heating	Kotikian et al. [45]	Moderate: Uses direct ink writing (DIW) for coaxial filaments with a liquid metal core. Requires complex core-sheath extrusion setup.	Supports consistent thermal response and mechanical stability in closed-loop operation.	Self-sensing via resistive feedback from liquid metal for deformation monitoring and closed-loop control.	Enables contractile and flexural actuation and integrates strain and actuation sensing functions.	Slow response due to thermal diffusion requires high current densities to facilitate actuation.
Laser-induced actuation	He et al. [46]	High: Electrospinning of precursor solutions with inherent alignment from electric fields; simple for small-scale production and avoids complex steps.	Fast response (300%/s strain rate) and power density (400 W/kg); suitable for micro-robotic durability.	Not explored.	High power density.	Modest actuation stress, non-conductive, and not capable of joule heating.
Joule heating	Wu et al. [28]	Moderate: Melt spinning for fibers with conductive coatings; multi-step but uses common techniques for alignment and curing.	High mechanical strength and repeatable cyclic actuation with negligible loss in performance.	Self-sensing via composites for closed-loop control in some variants.	Versatile multi-stimulus responses; large strains (up to 40–50%); integrates with photoelectric conversion.	Minimal constraints, but adhesion and robustness of MXene coating not assessed.
Photothermal and thermally responsive	Liang et al. [47]	Simple: Composite fabrication with MXene fillers; simple embedding via lamination or printing for dual-mode synergy.	Robust material for programmable deformations; fatigue resistance in morphing structures.	Integrated temperature/resistance feedback for environmental interaction.	High work density and strains (~40–60%); supports untethered operation with batteries.	Slower response (seconds) and a lack of ability for electro-responsive actuation.
Joule heating	Ince et al. [29]	Moderate: Multiple steps each spanning several hours; however, a simple wet-chemistry-based method is employed.	Electro-responsive actuation, strain sensing, and self-actuation sensing.	Integrated sensing functionalities.	Fast actuation response with integrated multi-functionality.	Employed method diminished performance and mechanical integrity.
Joule heating	Wang et al. [36]	Moderate: Novel method for producing ultra-thin LCE films followed by Au-sputtering.	Ultrafast actuation rate due to high conductive coating–LCE ratio.	Strain sensing and joule heating.	Ultrafast actuation rate.	Difficult to scale and limited to the production of LCEs with micron-scaled thickness.
Photothermal and thermally responsive LCEs	Hou et al. [48]	Moderate: Scalable bioinspired method for continuous production of long LCE fibers.	High actuation stress, strains, and work density.	Not applicable.	Ultrafast production rate; exceptional actuation performance.	Limited to actuating in response to thermal energy and IR radiation. Not capable of joule-heating.
Joule heating	Sun et al. [27]	Moderate: Roll-to-roll continuous production of LM LCE fibers.	LCE fibers with moderate to high tensile strength.	Not explored, but has potential for strain sensing.	Ultrafast actuation rate and long, continuously produced LCE fibers.	Laminating method could lead to reproducibility and scaling issues.
Joule heating	This work **	Moderate: Two-step, but utilized method employs resources inaccessible to most academic institutions (wet-chemistry and sputter coating).	Fast actuation (~1 s); robust adhesion of joule-heating coating to LCE.	Multi-functional LCEs: Strain sensing, self-actuation sensing, thermally responsive, and joule heating.	Provides a simple method for potentially improving adherence of various coatings to LCEs, opening the door for the development of future high-performance and multi-functional LCEs.	Non-continuous production method and multi-step fabrication method.

## Data Availability

Data are contained within the article and Supplementary Materials.

## Supplementary Materials

The following supporting information can be downloaded at: https://www.mdpi.com/article/10.3390/nano15211658/s1, Figure S1. Depicting the DAQ-DMA coupled set-up used to conduct the strain sensing and self-actuation sensing characterizations. Figure S2. Depicting the results to the conducted long-term cyclical strain sensing of the produced Au-PDA-LCEs. Video S1: No weight Au-LCE act video; Video S2: Au-LCE with weight video; Video S3: Au-PDA-LCE arm actuation video.
