# Supplementary material for: Robust and Multi-Functional Electrically Responsive Gold/Polydopamine-Coated Liquid Crystalline Elastomer Artificial Muscles
Source: Nanomaterials (Basel). 2025 Oct 31;15(21):1658. doi: 10.3390/nano15211658 (PMC12608711; doi:10.3390/nano15211658)
Supplement: Supplementary file 1 [file nanomaterials-15-01658-s001.zip › nanomaterials-3938021-supplementary.pdf]

Robust and Multi-functional Gold/Polydopamine Coated Liquid Crystalline Elastomers

Joshua C Ince [1], Alan R Duffy [2,3], Nisa Salim [1, 2, 3] *

[1] School of Engineering, Swinburne University of Technology, Hawthorn, Melbourne, Victoria 3122, Australia

[2] Centre for Astronomy and Supercomputing, Swinburne University of Technology, Hawthorn, VIC, 3122, Australia

[3] Space Technology and Industry Institute, Swinburne University of Technology, Hawthorn, VIC, 3122, Australia

[§] These authors contributed equally to this work.

* Corresponding author.

Nisa Salim, Tel: +61 392145703, E-mail: nsalim@swin.edu.au

The set-up employed to conduct the strain-sensing and self-actuation sensing characterizations is depicted in **Figure S1**. Two high temperature stable silicon insulated wires were connected to the DAQ via a 2-wires Ohmic resistance channel. The wires were then run up through the bottom DMA sample holding geometry, and down through the top DMA sample holding geometry. The top wire was then fixed to the top fixture using Kapton tape to ensure it was incapable of moving and the axial force on the DMA was tared to eliminate the force that the wire was adding to the upper holding geometry. Next the samples were fixed into the geometries and the wires were connected to the top and the bottom of the samples and fixed in place using conductive copper tape to achieve an electrical circuit where the current runs through the section of the sample that is being tested. The samples were then either mechanically strained or heated and the 2-wire electrical resistance was logged using the DAQ.

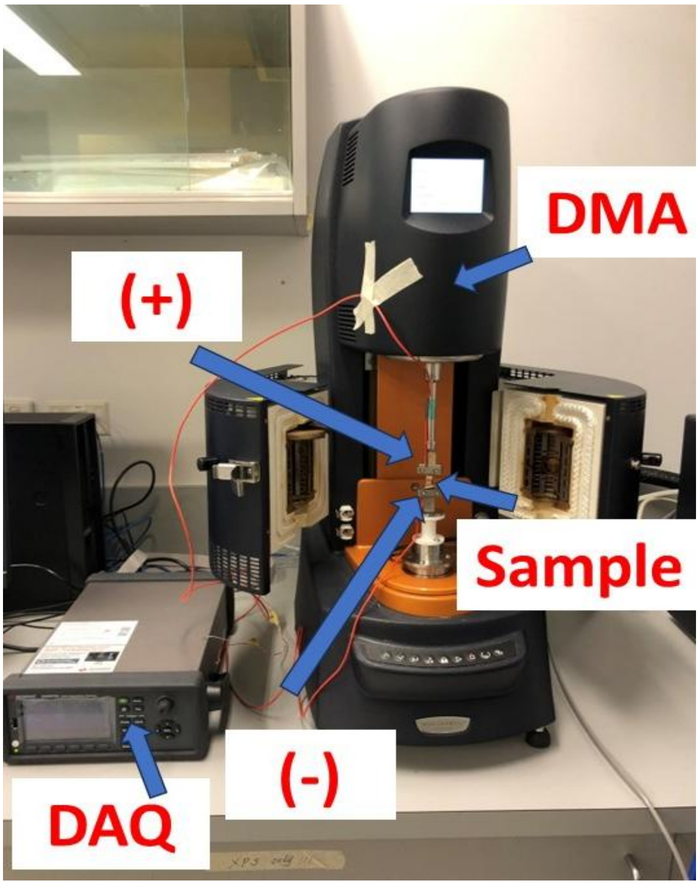

Figure S1. Depicting the DAQ-DMA coupled set-up used to conduct the strain sensing and self-actuation sensing characterizations.

Figure S2 depicts the results to the long-term cyclical strain sensing for the Au-PDA-LCE sample. The sample was cyclically mechanically strained and relaxed 100 times using the DMA/DAQ setup previously described and presented in **Figure S1**. As depicted the, the gain $\Delta\Omega/\Omega_0$ signal did track the cyclically applied mechanical strain. However, the sample was far from an effective strain sensor. Notably, not only was there evidence of creep (observable in the steadily increasing $\Delta\Omega/\Omega_0$ trough values), but also the peak heights seemingly spiked and varied in unpredictable ways. It's possible that this may be due to the inaccuracies of the 2-point probe coupled DMA testing method. However, without further investigations, we cannot assert the true cause of these results.

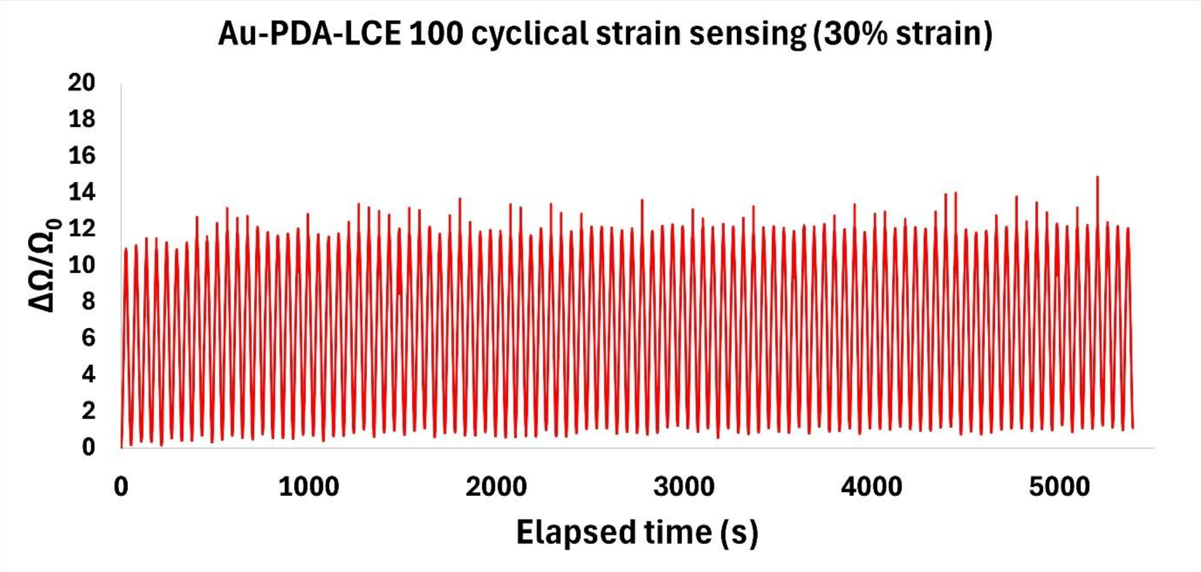

Figure S2. Depicting the results to the conducted long-term cyclical strain sensing of the produced Au-PDA-LCEs.
